# Determination of the adsorption isotherms and transport diffusivities of gases in mixtures inside zeolitic crystals using Infra-Red Micro-imaging

**DOI:** 10.1016/j.mex.2020.100993

**Published:** 2020-07-12

**Authors:** D. Carter, F.H. Tezel, B. Kruczek, J. Kärger, C. Chmelik

**Affiliations:** a University of Ottawa, Canada; b Leipzig University, Germany

**Keywords:** Zeolite characterization, Silicalite, Infra-Red Micro-imaging, Adsorption, Diffusion

## Abstract

Different regions of Infra-Red (IR) light absorption by guest molecules inside a zeolitic crystal are measured and quantified to determine binary adsorption isotherms and transport diffusivities. This has been achieved using a vacuum capable setup which includes an Infra-Red Microscope (IRM) and Fourier Transform Infra-Red (FTIR) Spectrometer. By utilizing IR light and FTIR spectroscopy, this method can be used to describe the behavior of low concentrations of relatively fast molecules inside zeolitic crystals as an alternative to chromatographic pulse methods. To demonstrate the capabilities of this method, binary adsorption isotherms and transport diffusivities of CO_2_ in mixtures composed of CO_2_ and N_2_ inside silicalite have been determined. From the fundamental measurements determined using this method, complex gas separation processes such as swing adsorption and multi stage membrane systems can be designed for novel zeolite materials. This method can also be used to develop models for complex adsorption and diffusion systems, and validate sophisticated molecular simulation models.

• IR microimaging with static gas dosing system for measuring transient uptake, diffusion and chemical reactions of gases and their mixtures in individual crystals or particles of nanoporous materials
• Using giant crystals the setup allows to study adsorption and transport of single components and mixtures in nanoporous materials also for fast diffusing guest molecules

## Graphical abstract

**Figure:**
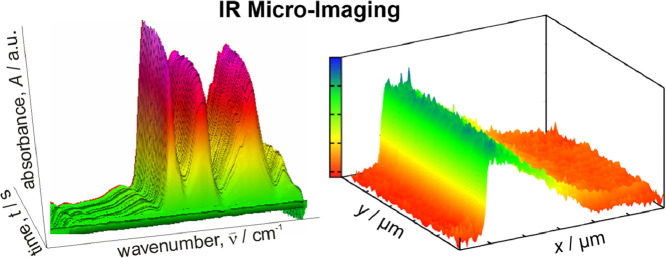

**Nomenclature**
**A**: Absorbance spectra
**APR 266**: Pressure transducer model
**CMR 361, 363**: Pressure transducer models
**CO_2_**: Carbon Dioxide
**FTIR**: Fourier Transform Infra-Red
**I**: Transmittance spectra
**I_0_**: Reference spectra
**IR**: Infra-Red
**IRM**: Infra-Red Microimaging
**N_2_**: Nitrogen
**PKR 251**: Pressure transducer model
**R**: Reference spectra
**T**: Transmittance spectra

## Specifications table

**Table:** 

**Subject Area**	***• Chemical Engineering OR*** ***• Physics and Astronomy***
**More specific subject area:**	Zeolite characterization
**Method name:**	Infra-Red Microimaging Analysis of Gases in Mixtures.
**Name and reference of original method**	C. Chmelik, L. Heinke, P. Kortunov, J. Li, D. Olson, D. Tzoulaki, J. Weitkamp, J. Kärger, Ensemble Measurement of Diffusion: Novel Beauty and Evidence, ChemPhysChem 10 (2009) 2623–2627.
**Resource availability**	Infra-Red Microscope (Bruker, Hyperion 3000) Fourier Transform Infra-Red Spectrometer (Bruker, Vertex 80V) Turbo molecular pump (Pfeifer, HiCube 80) Tubular furnace (Carbolite Gero Ltd, Carbolite MTF 12/38/400) High purity gases Zeolitic crystal

## Method details

### Equipment and materials

**Fig. 1:**
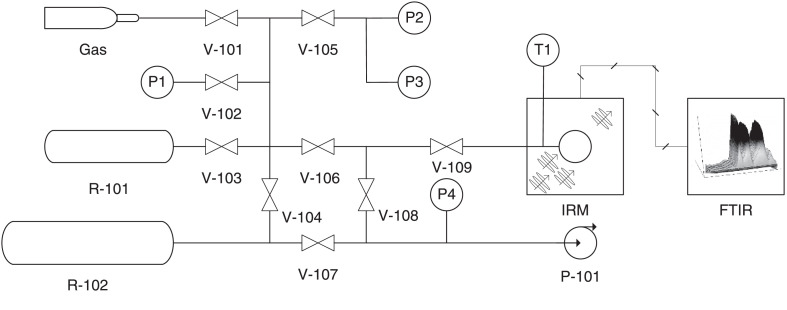
The schematic diagram showing the experimental setup that was used in this method. P1 – P4 are pressure transducers, and T1 is a K type thermocouple which can be used to measure room temperature in the vicinity of the quartz cell. V-101 - V-109 are gate valves. R-101 and R-102 are stainless steel reservoirs, and P-101 is a vacuum pump. The regeneration furnace is not shown. The pressure transducers are made by Pfeifer (Asslar, Germany) and have operating ranges of 0.1 – 1100 kPa (APR 266, accuracy ± 2% full scale), 1 × 10^−4^ – 1.1 kPa (CMR 363, accuracy ± 0.2% full scale), 1 × 10^−2^ – 110 kPa (CMR 361, accuracy ± 0.2% full scale), and 5 × 10^−8^ – 100 kPa (PKR 251, accuracy ± 30%) for P1 – P4 respectively. The gas reservoirs R-101 and R-102 are made from 316 stainless steel, and have capacities of 0.473 L and 3.785 L, respectively.

The experimental setup that was used in this method consists of two stainless steel gas reservoirs, several pressure transducers, a turbomolecular pump, and multiple gate valves that were connected to a quartz sample cell containing the zeolite crystal to be characterized. The quartz cell of this system was placed on the stage of an Infra-Red Microscope (IRM), which was connected to a Fourier Transform Infra-Red (FTIR) Spectrometer in order to measure the amount of Infra-Red (IR) light absorbed by the sample crystal under different experimental conditions. Details of this setup can be seen in Fig. 1. Additionally, convoluted vacuum tubing and VCO type fittings have been used so that the quartz cell can be removed from the stage of the IRM and placed inside a portable tubular furnace without disconnecting the quartz cell from the setup. The IRM and FTIR devices that were used in this method are the Hyperion 3000, and Vertex 80V models respectively, which are made by Bruker (Ettlingen, Germany). The portable furnace was a Carbolite MTF 12/38/400 made by Carbolite Gero Ltd (Neuhasen, Germany), and the turbomolecular pump was a Highcube 80 made by Pfeifer (Asslar, Germany).

### Experimental method

The following method has been used to characterize various combinations of zeolite crystals and gas mixtures, and has been developed from the work of Chmelik et al [1,2]. The diffusivities of large molecules which diffuse slowly in microporous materials (e.g. n- butane and i-butane) are typically measured using IRM, and so the novelty of the following method lies in its ability for determining the diffusivities of rapidly diffusing molecules such as CO_2_. The proposed method additionally enables the determination of adsorption behaviour as an alternative to volumetric, gravimetric, piezometric and chromatographic methods.

1. The zeolite crystal to be characterized was placed inside a quartz sample cell and connected to the experimental setup. The quartz cell was then placed inside the portable tubular furnace, valves V-101 to V-109 were opened, and the system was evacuated using vacuum pump P-101. During evacuation, the quartz cell was subjected to an appropriate temperature for the removal of moisture from the crystal (which is dependent on the crystal type, and is typically in the range of 400°C – 450°C). Once evacuated, the quartz cell was removed from the portable oven and placed onto the stage of the IRM. V-101 to V-109 were then closed.
2. For pure gas uptake experiments, reservoir R-102 was then filled from the gas cylinder up to the desired maximum experimental pressure as measured by P1 by opening V-101, V-102, and V-104. An additional amount of gas was then added to R-102 in order to compensate for the system volume at the maximum pressure. V-101, V-102 and V-104 were then closed. For gas mixture uptake experiments at room temperature, ideal gas behavior has been assumed, and the partial pressure of each gas as a fraction of the total gas pressure was manipulated in order to fill R-102 with a predetermined molar composition. Since there is only one sample gas port in the setup shown in Fig. 1, it was necessary to re-evacuate the process lines after the second cylinder had been connected to the system. This was done by opening V-101, V-106, and V-108. Once the process lines were re-evacuated, V-101, V-106, and V-108 were closed.
3. Gas uptake experiments to determine transport diffusivities were then conducted according to the following procedure:
  3.1. The IRM was first operated in optical mode, and its IR laser was focused on the zeolite crystal. The IRM was then switched to IR transmittance mode, and the IR transmittance spectrum of the crystal was determined. This spectrum was the reference spectrum.
  3.2. Reservoir R-101 was filled with gas from R-102 by opening V-103 as well as V-105, and then slowly opening V-104 until the desired pressure was reached. V-104 was then closed. For a step change in pressure from 0 to 10 kPa, R-101 was filled with gas from R-102 until the pressure measured by P3 was observed to be 10.2 kPa (where 0.2 kPa of pressure was required to compensate for the sample cell volume in order to reach a final pressure of approximately 10 kPa in the system).
  3.3. The FTIR was set to continuously determine IR transmittance spectra of the bulk gas phase and crystal, which was subjected to a step change in pressure by quickly opening V-109. Spectra were continuously determined until equilibrium had been realized, which was confirmed by observing that the IR transmittance spectra did not change with time. These spectra were analyzed subject to the reference spectrum determined in step 3.1.

4. The amounts of gas adsorbed by the zeolite crystal at equilibrium were then determined according to the following procedure:
  4.1. The FTIR was used to determine the IR transmittance spectra of the bulk gas phase and crystal at equilibrium.
  4.2. The IRM was switched back to optical mode, and the quartz cell was translocated on the IRM stage such that the IRM laser was focused on a region of the quartz cell where no crystal was present. The IRM was then switched back to IR transmittance mode, and the IR transmittance spectra of this space, which describes the bulk gas phase only, were determined. The equilibrium spectra determined in step 4.1 were analyzed given the reference spectra of both the crystal as determined in step 3.1, and the bulk gas phase as determined in this step; step 4.2.
  4.3. The IRM was again switched back to optical mode, and the quartz cell was translocated back to its original location such that the IR laser was focused on the zeolite crystal as before. V-109 was then closed.

5. Steps 3 and 4 were repeated for all pressure steps of interest.

### Analysis of experimental data

From the IR transmittance spectra (I) that were obtained in steps 3 and 4, IR absorption spectra (A) were determined. This has been achieved by dividing the reference spectrum (I_0_) by each transmittance spectrum, and then taking its logarithm in accordance with the Beer-Lambert law [3], as described by Eq. (1) [4]. Given IR absorption spectra, the molecular bonds of guest molecules and zeolitic crystals can be distinguished from each other according to their different bands in IR absorbance spectra. Molecules with dissimilar IR spectra (including isomers) can therefore be distinguished from each other using IRM, and this method can be used to determine binary and possibly multicomponent concentrations depending on the characteristics of the gases and the crystal. These different bands can be observed as IR absorption spectra peaks, and were quantified by integration.

(1) $$A=log\left(\frac{I_{0}}{I}\right)$$

In order to determine transport diffusivities from IRM data, the previously published method by Chmelik et al has been used [1,2]. In this method, time dependent IR absorbance spectra peaks from step 3 corresponding to the guest molecules of interest have been integrated. Relative uptake as a fraction of complete uptake was then determined based on these integrals, and was used in combination with an appropriate analytical expression in order to find the transport diffusivity of the guest molecules by regression analysis. Analytical expressions which describe the diffusion of guest molecules inside different shapes such as slabs, spheres, and cylinders have been published by Crank and can be applied to appropriately shaped crystals as required [5].

In order to generate binary adsorption isotherms from experimental data using IRM, the relationship between guest molecule IR absorbance and molar gas concentration was correlated to generate a calibration curve. For ideal systems, a single literature adsorption isotherm value can be used to generate the required calibration curve in accordance with the Beer-Lambert law, which describes absorbance as a linear function of path length and concentration [3]. For non-ideal systems, IR absorption spectra peak integrals pertaining to the guest molecules at numerous pressures can be correlated to literature isotherms to generate the required calibration curve.

### Method validation

This method has been validated using a crystal of silicalite with pure CO_2_ gas, and gas mixtures composed of CO_2_ and N_2_. In this investigation, the silicalite crystal was cuboid shaped, and had height, width, and length dimensions of 510 µm, 490 µm, and 1200 µm, respectively. This crystal was fabricated according to a similar method to the one published by Kida et al using quartz as the silica source [6]. The CO_2_ and N_2_ gases that were used for pure gas and gas mixture experiments had purities of 99.998%, and were purchased from Sigma (Schnelldorf, Germany). Adsorption isotherms and transport diffusivities were found for CO_2_ but not N_2_ since it does not absorb IR light, and so cannot be detected using IRM. These results have been published elsewhere [7]. Isotherms for pure CO_2_ and silicalite determined using other methods have been published by others [8], [9], [10], [11], which are in agreement with the isotherms generated in this study, and confirm that this method is reliable. As a result, the binary adsorption isotherms for CO_2_ can also be considered reliable since their IR absorbance spectra is distinct from that of N_2_.

Order of experiments and crystal regeneration conditions for silicalite and mixtures composed of CO_2_/N_2_ were as follows:

I. The zeolite crystal was first regenerated by removing all adsorbed species from its structure under a vacuum pressure of less than 10^−4^ mbar at a temperature of 450°C for 11 hours as described in step 1 of the experimental method section. Heating and cooling temperature rates of 5°C per minute were used.
II. A gas mixture composed of 15% / 85% CO_2_ / N_2_ was prepared, and gas uptake and amounts adsorbed at equilibrium were investigated for CO_2_ at pressure steps of 10 kPa from 0 to 100 kPa, and then pressure steps of 25 kPa from 100 kPa to 250 kPa. Pure N_2_ was not investigated due to its poor absorption of IR light at the experimental conditions.
III. The zeolite crystal was regenerated at room temperature by removing all adsorbed species from its structure under a vacuum of less than 10^−4^ mbar.
IV. Step II and then III were repeated in turn for gas mixtures composed of 30% / 70%, 50% / 50%, 70% / 30%, 85% / 15% CO_2_ / N_2_, and then 100% CO_2_.

Two gas phase compositions could be tested in a single day, and so three days were required to completely characterize the zeolite crystal in this way. To ensure that there was no contamination of the crystal when the crystal was not being characterized (during the night), the crystal was regenerated as described in step I overnight.

**Fig. 2:**
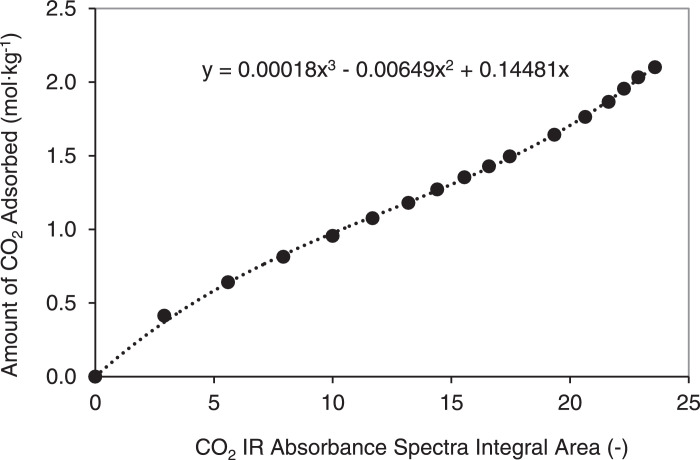
Calibration curve describing the relationship between experimentally determined IR absorbance spectra which were integrated between wavelengths of 3550 and 3740 cm^−1^ for CO_2_, and the amount of CO_2_ adsorbed on silicalite. IR absorbance spectra were determined from experiments that were conducted at pressures between 0 kPa and 250 kPa using pure CO_2_. The amounts of CO_2_ adsorbed on silicalite have been taken from the isotherm data obtained by Li and Tezel [11]. Temperature effects have been accounted for by utilizing the temperature dependent Sips model for isotherm data at a temperature of 32°C.

**Fig. 3:**
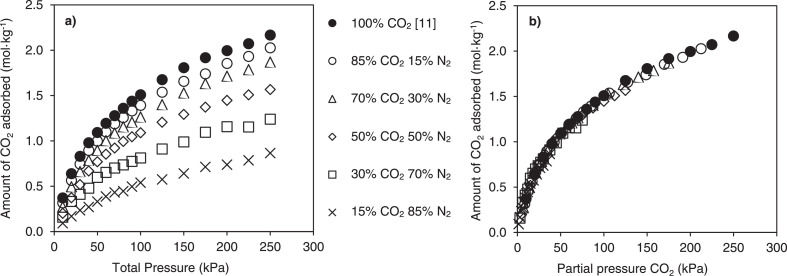
Amount of CO_2_ adsorbed at equilibrium on a single crystal of silicalite when present as a pure gas, and in binary CO_2_ and N_2_ mixtures, as a function of a) total pressure, and b) partial pressure. The experimental uncertainty is smaller than the symbol size. All these experiments were conducted at 32 ± 2°C.

**Fig. 4:**
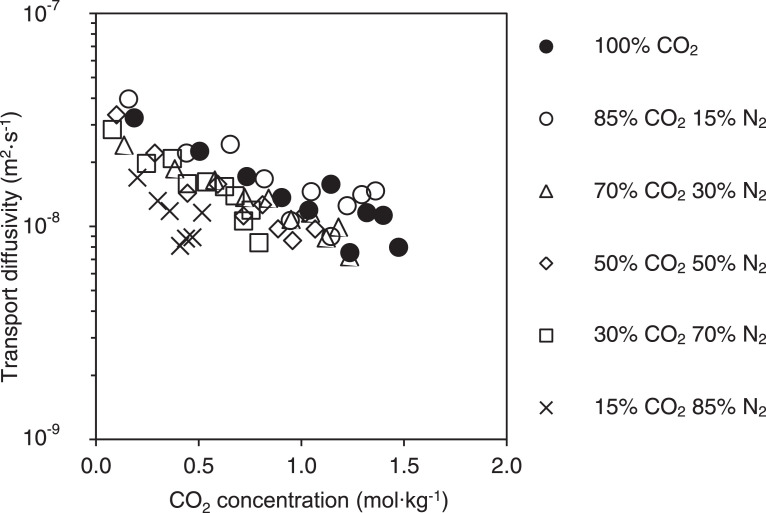
Apparent CO_2_ transport diffusivities expressed as a function of CO_2_ concentration in the adsorbed phase. The experimental uncertainty is about twice the symbol size. Concentration in this figure refers to the average amount of CO_2_ adsorbed between the initial and final pressures of the uptake experiment. Experiments were conducted at a temperature of 32 ± 2°C and total pressures between 0 and 100 kPa using a range of mixture compositions. The crystal was assumed to be cuboid shaped and infinitely long in order to determine transport diffusivity by regression analysis. The height, width, and length dimensions of the crystal were 510 µm, 490 µm, and 1200 µm respectively.

Figs. 2–4 show the calibration curve, binary adsorption isotherms, and transport diffusivities for CO_2_ inside silicalite at various compositions and pressures with N_2_ in silicalite, respectively.

## Declaration of Competing Interest

The authors declare that they have no known competing financial interests or personal relationships that could have appeared to influence the work reported in this paper.

## References

[1] Chmelik C., Varma A., Heinke L., Shah D.B., Kärger J., Kremer F., Wilczok U., Schmidt W. (2007). Effect of surface modification on uptake rates of isobutane in MFI crystals: An infrared microscopy study. Chem. Mater..

[2] Chmelik C., Heinke L., Kortunov P., Li J., Olson D., Tzoulaki D., Weitkamp J., Kärger J. (2009). Ensemble measurement of diffusion: Novel beauty and evidence. ChemPhysChem.

[3] Swinehart D.F. (1962). The Beer-Lambert Law. J. Chem. Educ..

[4] Kärger J., Ruthven D.M., Theodorou D.N. (2012). Diffusion in Nanoporous Materials.

[5] Crank J. (1975). The Mathematics of Diffusion.

[6] T. Kida, K. Kojima, H. Ohnishi, Synthesis of large silicalite-1 single crystals from two different silica sources, 30 (2004) 727–732. doi:10.1016/j.ceramint.2003.08.011.

[7] Carter D., Tezel F.H., Kruczek B., Kärger J., Ruthven D., Marthala R., Chmelik C. (2020). Equilibrium isotherms and transport diffusivities for CO2 and CO2/N2 mixtures in silicalite measured by Infra-Red Micro-imaging. Microporous Mesoporous Mater.

[8] Rees L.V.C., Bruckner P., Hampson J. (1991). Sorption of N_2_, CH_4_ and CO_2_ in Silicalite-1. Gas Sep. Purif..

[9] Sun M.S., Shah D.B., Xu H.H., Talu O. (1998). Adsorption Equilibria of C_1_ to C_4_ Alkanes, CO_2_, and SF_6_ on Silicalite. J. Phys. Chem. B..

[10] Dunne J.A., Rao M., Sircar S., Gorte R.J., Myers A.L. (1996). Calorimetric heats of adsorption and adsorption isotherms .2. O_2_, N_2_, Ar, CO_2_, CH_4_, C_2_H_6_, and SF_6_ on NaX, H-ZSM-5, and Na-ZSM-5 Zeolites. Langmuir.

[11] Li P., Tezel F.H. (2008). Pure and binary adsorption of carbon dioxide and nitrogen on silicalite. J. Chem. Eng. Data..

